# Immunotherapeutic Approaches for the Treatment of Glioblastoma Multiforme: Mechanism and Clinical Applications

**DOI:** 10.3390/ijms241310546

**Published:** 2023-06-23

**Authors:** Suprava Das, Banendu Sunder Dash, Thejas P. Premji, Jyh-Ping Chen

**Affiliations:** 1 Department of Chemical and Materials Engineering, Chang Gung University, Kwei-San, Taoyuan 33302, Taiwan; supravadas0603@gmail.com (S.D.); banendusunder@gmail.com (B.S.D.); thejaspremji@gmail.com (T.P.P.); 2 Department of Neurosurgery, Chang Gung Memorial Hospital at Linkou, Kwei-San, Taoyuan 33305, Taiwan; 3 Craniofacial Research Center, Chang Gung Memorial Hospital at Linkou, Kwei-San, Taoyuan 33305, Taiwan; 4 Research Center for Food and Cosmetic Safety, College of Human Ecology, Chang Gung University of Science and Technology, Kwei-San, Taoyuan 33305, Taiwan; 5 Department of Materials Engineering, Ming Chi University of Technology, Tai-Shan, New Taipei City 24301, Taiwan

**Keywords:** glioblastoma multiforme, brain cancer, clinical application, immunotherapy

## Abstract

Glioma is one of the most aggressive types of primary brain tumor with a high-grade glioma known as glioblastoma multiforme (GBM). Patients diagnosed with GBM usually have an overall survival rate of less than 18 months after conventional therapy. This bleak prognosis underlines the need to consider new therapeutic interventions for GBM treatment to overcome current treatment limitations. By highlighting different immunotherapeutic approaches currently in preclinical and clinical trials, including immune checkpoint inhibitors, chimeric antigen receptors T cells, natural killer cells, vaccines, and combination therapy, this review aims to discuss the mechanisms, benefits, and limitations of immunotherapy in treating GBM patients.

## 1. Introduction

**Figure 1:**
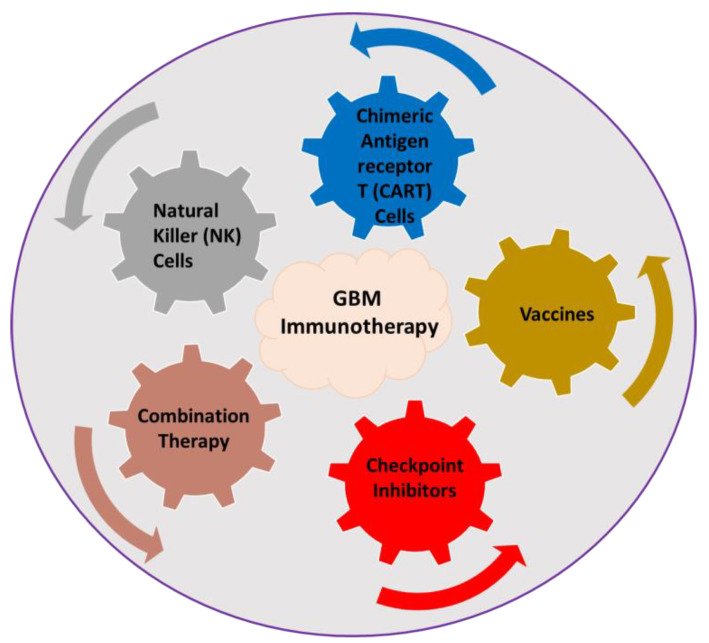
The immunotherapeutic approaches currently used for treating glioblastoma multiforme (GBM).

There are numerous strategies developed over the years for the treatment of fatal brain cancer diseases. Amidst all the frontline treatments for brain cancer, the goal is to achieve quick recovery with minimally invasive surgery and fewer skin incisions. The glioblastoma multiforme (GBM) is a highly invasive brain cancer in humans. The median survival of patients diagnosed with GBM, mainly in adults, is typically less than 1.5 years despite chemotherapy and radiotherapy, and the average 60-month survival is only 10% [1,2,3]. These poor outcomes are primarily due to the proliferation of tumor cells into the surrounding tissue, which hinders the traditional therapeutic approaches. In light of these facts, immunotherapy might be a promising therapeutic modality. Cancer immunotherapy, in its broadest sense, refers to a treatment based on the methods used by the immune system to eliminate cancerous cells. To stimulate or improve the capacity of endogenous immune effector cells to target and destroy tumor cells, immunotherapy comprises the administration of different interleukins, cytokines, and chemokines. Recently, several types of immunotherapeutic approaches are being studied for treating GBM, including immune checkpoint inhibitors, chimeric antigen receptors T (CAR T) cells, natural killer (NK) cells, vaccines, and combination therapy (Figure 1) [4,5,6,7,8]. However, the trafficking of the right kind of immune cells from the periphery into the brain remains one of the major challenges for immunotherapy of GBM [9]. Because the vascular components and immune cells in the TME will change their function and properties, this may create a major hurdle toward the successful treatment of brain tumors. The tumor-associated microglia and macrophages (TAMs) can highly infiltrate the GBM, which can support the glioblastoma cells and promote tumor progression. During tumor growth, the vasculature changes dramatically to impede the upregulation of many growth factors, including the vascular endothelial growth factor (VEGF) that is responsible for angiogenesis [10]. Traditional treatments for most primary and secondary GBM include surgery, chemotherapy, and/or radiation therapy. Eventually, these methods can combine with laser-based therapy or non-laser-based ablative approaches. However, the endothelial blood–brain barrier (BBB) forms a barrier between the blood and the central nervous system (CNS) to obstruct the systematic delivery of therapeutic agents into the brain, where the efficiency of administrating therapeutic agents depends on features like molecular weight, size, surface charge, nature of the drug carrier, solubility of drugs, retention time, and stability of the drug [11,12]. For GBM, immunotherapy has garnered a lot of attention since it can trick the immune system into attacking tumor cells while causing the fewest side effects possible. The absence of conventional lymphatics in the CNS makes it immune-privileged by restricting the entry of immune cells [13]. However, activated T cells in the cervical lymph nodes can enter the brain parenchyma through the cerebrospinal fluid (CSF). The microglia, which are immune cells localized in the brain, can also serve as potential antigen-presenting cells (APCs). Some studies found that blood-derived immune cells are not entirely excluded from the brain and that the brain is immunologically privileged in certain respects [14,15,16,17]. The high-molecular-weight polar proteins like growth factors, chemically modified enzymes, immunoglobulins, conjugates of proteins, and genetically engineered viral vectors are the most used therapeutic interventions [18]. 

The two main issues for treating glioblastoma are its resistance to conventional therapies and frequent recurrence. The nano-therapeutic approaches have been used to overcome the BBB for glioblastoma treatment [19]. Other than this, immunotherapies may be another promising treatment modality. Numerous immune-based therapeutic approaches have been proposed and used in clinical studies, alone or in combination. Therefore, this review tries to elucidate all strategies regarding immune-based treatments of GBM with their mechanism, and in combination with other therapies. We will discuss the advantages and disadvantages of using immunotherapy to treat GBM.

## 2. Role of Blood–brain barrier (BBB) in Brain Cancer Treatment

**Figure 2:**
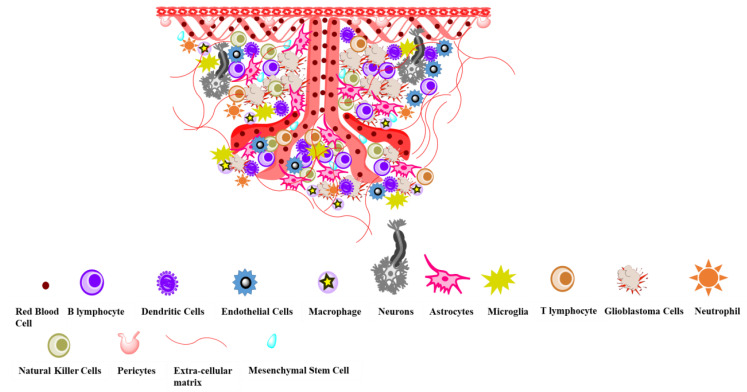
Tumor microenvironment (TME) in glioblastoma multiforme (GBM).

The BBB plays an important role by providing a microenvironment for generating neuronal signals. Its anatomy is composed of endothelial cells, basal lamina, and astrocytes. In the CNS, the proper function of vasculature is required to supply oxygen and nutrients, as well as to inhibit the efficiency of immunological responses and to prevent the access of immunoglobulins, leukocytes, cytotoxic substances, peptides, and drugs from the brain interstitial space. It has been found that 98% of low-molecular-weight drugs, and all high-molecular weight therapeutic agents such as recombinant proteins, antibodies, and viral vectors, have failed to cross the BBB [20]. The tumor microenvironment (TME) of GBM constitutes several components, including astrocytes, macrophages, neurons, and endothelial cells (Figure 2). The endothelial cells can induce necrotic cell death while simultaneously facilitating the downregulation of the immune cell-mediated inflammatory response [21]. While endothelial cells of brain capillaries are the principal cellular element of BBB, the structural integrity of BBB requires the close association and interaction of endothelial cells with astrocytes, one of the major non-neural cells in the brain during angiogenesis and BBB development [22]. During BBB development, the pericytes and endothelial cells can bind with several growth factors like VEGF, transforming growth factor, or platelet-derived growth factor [23]. On the other hand, sprouting or splitting from pre-existing vessels forms new capillaries known as brain vascularization, during which excess endothelial cells are generated. The vascular sprout will migrate towards tumor-secreted angiogenic growth factors (e.g., VEGF) and cause tumor angiogenesis during the growth of brain tumors [24]. These growth factors can bind with their respective surface receptors expressed on the surface of endothelial cells. Therefore, to inhibit endothelial proliferation and migration, anti-angiogenic agents can be used to delay the VEGF-induced proliferation and migration of endothelial cells [25,26]. 

The ligand-mediated drug delivery strategy can deliver drugs across the BBB by conjugating transporting ligands to drug delivery vehicles, whose receptors are highly expressed on the BBB endothelial cells. This can be also accomplished via carrier-mediated delivery, receptor-mediated delivery, vector-mediated delivery, and stem cell-mediated delivery [27,28]. In ligand-mediated delivery, the drug delivery vehicles can be associated with endogenous ligands, such as insulin transferrin, folic acid, and lectins, which show a high affinity towards brain tumor cells. Common ligand-induced endocytosis can also downregulate the signals generated from the growth factors [29]. The carrier-mediated delivery accomplishes the transport of molecules, such as glucose, amino acids, fatty acids, minerals, and vitamins across the BBB by binding to their corresponding transporter proteins. For example, sodium glucose-linked transporters (SGLTs) can facilitate the transport of glucose without utilizing adenosine triphosphate (ATP) [30]. The other transport mechanism across the BBB is receptor-mediated delivery, which is comprised of several steps. The first step involves the circulation of a ligand linked with its corresponding transmembrane receptor expressed on the apical plasma membrane. In the second step, endocytosis occurs through membrane invagination, followed by the formation of intracellular vesicles conjugated with receptor-ligand complexes. The next step involves the trafficking of cellular vesicles, where vesicles can reach the targeted area crossing the BBB. In the last step, depending on the routes of the vesicles released area (basolateral or brain parenchyma), either transcytosis or exocytosis can occur. In vector-mediated delivery, vectors expressing tumor suppressor genes can alter the local TME [31]. Generally, vector-mediated delivery is classified into two types, viral-based vector delivery and non-viral based. Viral-based vector delivery is associated with the transport of genetically engineered virus genomes or foreign DNA into the cell, whereas non-viral-based vector delivery is accomplished with the transport of DNA into the nucleus of a cell by surface modification of DNA. Finally, stem cell-based delivery can use mesenchymal stem cells to regulate endothelial cell permeability and BBB integrity [32]. Apart from transport limitations in the BBB, the physiological features of CNS may create hurdles in the path of successful immunotherapy.

## 3. Immune Privilege of the Brain and the Prospect of Immunotherapies

In the context of immune-privileged organs, the brain is one of the most protected organs from immune cell entry and attack [33]. The macrophages are one of the major immune cells to maintain the integrity of CNS throughout the lifetime. The CNS is composed of a distinct population of mononuclear phagocytes with microglia being a part of this population, which guards the CNS from neuroinflammation that can induce immune responses. Microglia grow from the yolk sac through “hematopoiesis” during embryonic development and can cause phagocytosis during the CNS injury [34]. The macrophage originating from embryonic precursors can have distinct phenotypes and functions. Nonetheless, the phenotype of macrophages can be dramatically shifted in various processes, such as fibrosis and hypoxia, depending on the properties of the tumor. The colony-stimulating factor-1 (CSF1) and chemokine (C-C motif) ligand 2 (CCL2) are two major promoters for macrophage recruitment in the TME, while hypoxia can also induce the production of CSF1 and CCL2 by tumor cells, leading to the downregulation of the corresponding receptors [35]. The neuropilin-1(Nrp-1) is used as a precursor receptor molecule to prohibit the entry of TAMs into the hypoxic region by inhibiting the migration towards its ligand Sema3A, which can help to restore anti-tumor immunity [36]. 

**Figure 3:**
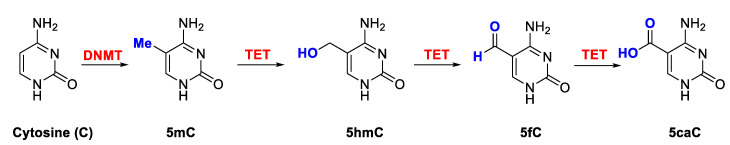
DNA modification as an epigenetic mechanism of gene expression regulation.

The enzymatic modification of histone proteins and DNA can lead to an altered epigenetic profile and specific gene expression patterns, leading to phenotypic variability. In the enzymatic modification of histone proteins, the N-terminal tails of histone proteins can be modified through acetylation, methylation, or phosphorylation. The DNA modification occurs by covalent modification of DNA through methylation, during which a methyl group is conjugated to a base in DNA. After DNA methylation, N1-methyladenine (m1A) and N3-methylcytosine (m3C) can be generated, which results in DNA damage [37]. The DNA methylation can also occur at the fifth carbon atom of a cytosine (C) ring with the help of a DNA methyltransferase (DNMT) within a cytosine-phosphate-guanosine (CpG) site to form 5-methyl cytosine (5mC) (Figure 3) [38]. The ten-eleven translocation (TET) enzyme oxidizes 5mC to 5-hydroxymethylcytosine (5hmC) and subsequently to 5-formyl cytosine (5fC) and 5-carboxyl cytosine (5caC), as shown in Figure 3. Specifically, the GBM patients have a genomic mutation pattern, and *IDH1, IDH2, PTEN, NARS, TP53, CDKN2A,* and *EGFR* are the emerging genes that frequently mutated in such cases [39]. Based on this mutation pattern, the transcriptional subclass of GBM has been subdivided into four groups classical, proneural, mesenchymal, and neural.

Due to the diversity of the mechanisms and complex signaling pathways, the basic treatment modalities for most frontline cancers involve either surgery or chemotherapy or are combined with radiation therapy. For GBM, the chances of recurrence cannot be avoided and the overall survival after the treatments is limiting. In neuro-oncology, it is very challenging to find the right treatment that can increase the survival rate and destruct all tumor cells without affecting healthy cells. Immunotherapy represents a safe and promising approach because of its antigen-targeting concepts. In 1893, William Coley pioneered the concept of immunotherapy by inoculating *streptococcus* in a sarcoma patient [40]. To establish antigen-targeting-based treatments with longer overall survival rates, the TME, which comprises various kinds of cell types with different functions and activities, needs to be focused on. The antigen-presenting cells (APCs) mainly belong to the major histocompatibility complex I and II (MHC- I and II). Checkpoint inhibitors have been found to downregulate T-cell activation. Considering this, the role of various checkpoint regulators, such as PD-1, CTLA-4, IDO-1, and LAG-3 for the prevention of autoimmunity, will be analyzed in detail in the following sections. To date, vaccines and virus-based therapies have been investigated for GBM treatment and proved to be a safe intervention. Oncolytic viruses are used as vectors for delivering signals, which is helpful to activate the adaptive immune system through pathogen-associated molecular patterns. Furthermore, it is widely accepted that CAR T cell-based therapy is an emerging approach toward the treatment of GBM, which is based on genetically modified T lymphocytes with a novel chimeric antigen receptor. Therefore, details of this approach will be also discussed further. Early approaches to adoptive cell therapy also involved NK cell-based therapy for GBM patients, which can prevent pathogen-associated inflammations. The molecular biomarkers may also solve the diagnosis and imaging problems that arise due to the molecular heterogeneity of GBM. All of these will be discussed further in the following sections.

### 3.1. Immune Checkpoint Inhibitors

**Figure 4:**
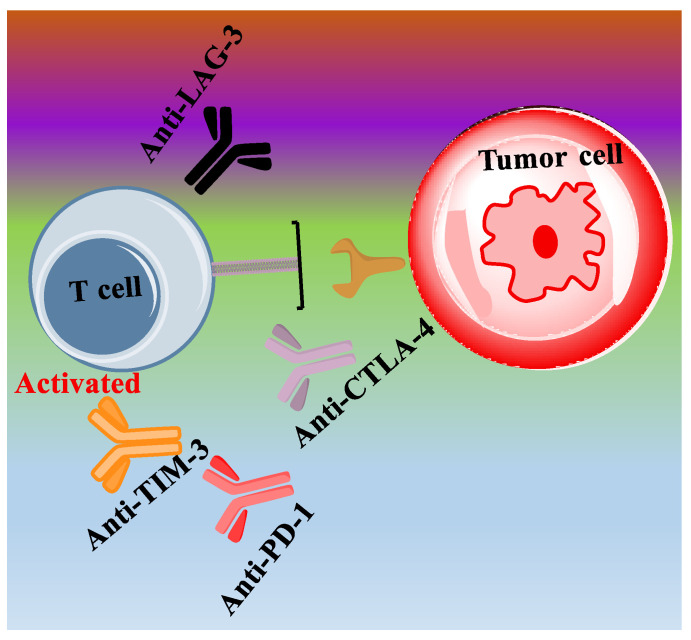
The T-cell exhaustion mediated by immune checkpoint protein PD-1, CTLA-4, TIM-3 or LAG-3 is inhibited using antibodies (anti-PD-1, anti-CTLA-4, anti-TIM-3 or anti-LAG-3) as immune checkpoint inhibitors.

Cancer treatment using immune checkpoint inhibitors is gaining increased popularity in clinical practice recently. As commonly known, proteins on the surface of T cells can recognize and bind to partner proteins on tumor cells, which are immune checkpoint proteins. Immune checkpoints engage when proteins on the surface of T cells recognize and bind to partner proteins on tumor cells, sending an “off” signal to T cells to prevent the immune system from destroying the tumor cells [41]. Immune checkpoint inhibitors, such as the antibody against PD-1, CTLA-4, TIM-3, or LAG-3 protein, can block the checkpoint proteins from binding with their partner proteins, which can prevent the “off” signal from being sent and allow T cells to kill cancer cells by alleviating the inhibitory effect (Figure 4) [42]. While it is unknown if immune checkpoint inhibitors can cross the BBB, animal models show that they have limited penetration [43]. It should be noted that the release of tumor- or host-specific cytotoxic T lymphocytes might elicit both activity and toxicity. Meanwhile, studies have demonstrated that resistance can arise at any stage of a tumor’s immunological response. Therefore, the mechanism of tumor-derived resistance caused by different immune checkpoints, as well as by T-cell exhaustion, needs careful study. In the context of T cell-based resistance and TME-determined resistance, cytotoxic T lymphocyte-associated antigen 4 (CTLA-4) and programmed death (PD-1) checkpoints are co-inhibitory (antagonistic) by inhibiting the immune responses and the antitumor responses [44]. Similarly, co-stimulatory receptors (agonistic) like OX40 and CD40 have been used in clinical trials [45]. Various preclinical studies have demonstrated the therapeutic benefits by combining these co-inhibitory and co-stimulatory molecules with the immune checkpoint blockade for eliciting optimal anti-tumor T cell activation. Recently, checkpoint inhibitors have received the most attention for immunotherapy from clinical trials, in which they have shown promising preclinical data. The clinical trials taken from clinicaltrials.gov using an immune checkpoint inhibitor (CTLA-4, PD-1, TIM-3, LAG-3 or IDO-1) for GBM treatment are included in Table 1. 

#### 3.1.1. Cytotoxic T-Lymphocyte-Associated Protein-4 (CTLA-4)

As a type 1 transmembrane glycoprotein, CTLA-4 was identified in 1995 by Krummel and Allison [46]. It is an immunological checkpoint protein having a deprecatory influence on self-tolerance and T-cell homeostasis for autoimmunity [47]. As it acts as a negative regulator of immunity, antibody-based blockade of CTLA-4 can help to achieve antitumor immunity. Initially, a CTLA-4-based therapeutic intervention is primarily focused on leveraging its immune deregulation in autoimmune diseases. However, the precise process behind this suppressive effect is currently unknown. The CTLA-4-mediated immunosuppression in GBM was reported to be associated with the infiltration of macrophages in the TME [48]. The CTLA-4 receptor, expressed on the surface of antigen-presenting cells such as regulatory T cells, competes with CD28 for binding to co-stimulatory molecules (CD80 and CD86) on these cells, which results in the immune system not being able to kill tumor cells efficiently. This arises from a reduction in the incidence of cancer lesions in the body [49]. An evaluation of tumor-infiltrating lymphocytes (TILs) in GBM patients indicates that the ratio of immunosuppressive T regulatory cells to effector T cells increases in these patients, and that CTLA-4 expression is increased in these T regulatory cells. This gives rise to an immune checkpoint blockade approach using the monoclonal antibody ipilimumab, which can target CTLA-4 to prevent its interaction with the ligands (B7.1 and B7.2) for enhancing the immune response [50]. However, the drug has only been approved as a treatment for renal cell carcinoma in the U.S., and for melanoma in the rest of the world so far [51]. Tremelimumab is another drug in this category, which mainly targets the CTLA-4 as well to help the immune system fight against malignancies. It has also been the subject of many clinical trials against different cancers. Although numerous trials are still being conducted, no promising results have yet been reported [52]. Recently, it has been proposed that tumors can use the same mechanisms to avoid immune system detection when they resist the therapeutic immune checkpoint blockade. Immune therapies using ipilimumab and tremelimumab would not function as effectively as they could due to this so-called immune editing. However, various research outcomes indicate that bringing different treatment modalities into one single platform may help to resolve this resistance issue. Considering another key checkpoint other than CTLA-4, PD-1 is one of the emerging targets in immunotherapy with the ligand PD-L1/L2 in the CD28 family. The clinical trial study NCT02794883 reported the use of tremelimumab and durvalumab (MEDI4736), a new class of drug based on an antibody targeted the PD-1, for phase 2 glioblastoma treatment. The tremelimumab can block the inhibitory signal resulting from the binding of CTLA-4 to B7 ligands on the surface of the APCs to prolong and enhance T-cell activation. However, poor prognosis ability was reported as per the outcome of the trial (Table 1). Although the outcomes may warrant further investigation into the use of anti-CTLA-4 treatments, their adverse effects which are related to renal function and gastrointestinal activity should be taken into consideration in the future. 

#### 3.1.2. Programmed Cell Death Protein-1 (PD-1)

The programmed death-1 (PD-1) is a type I membrane protein with 288 amino acids. It is an inhibitory receptor protein expressed on the T-cell surface and plays a role in regulating the immune system’s response by down-regulating the immune system through suppressing T-cell inflammatory activity [53]. After conjugation with the ligand PD-L1, PD-1 can be activated to recruit a tyrosine phosphatase, the Src homology phosphotyrosyl phosphatase 2 (SHP-2), which is involved in T-cell receptor CD28 signaling. With the de-phosphorylation reaction, the attenuation of key molecules in the CD28 pathway occurs, leading to the exhaustion of activated T cells, and finally, tumorigenesis takes place. To prevent damage to tissue and preserve self-tolerance, the human body evolves co-inhibitory pathways, but on the other hand, tumor cells block these inhibitory pathways to avoid host-immune surveillance by overexpressing PD-L1. PD-L1 is consecutively expressed at a low level of antigen-presenting cells (APCs), whereas PD-L2 is expressed on dendritic cells (DCs) and macrophages. Activation of PD-1 receptors on T cells, in particular, results in hyporesponsive/exhausted effector T cells. Immunotherapy has gained momentum in the recent decade due to the emergence of antibodies that suppress the activation of self-restricting immunological checkpoint receptors on T cells. Treatment of exhausted tumor-reactive T cells with ipilimumab, nivolumab, and pembrolizumab, in particular, stimulated the anti-tumor responses of T cells and demonstrated therapeutic efficacy in treating metastatic melanoma. The GBM, however, has a low initial mutational burden compared to immunogenic tumors. A phase 1 clinical trial examined the immune checkpoint inhibition with nivolumab for newly diagnosed O6-methylguanine–DNA methyltransferase (MGMT)-methylated GBM (NCT02667587), but it was canceled later due to poor outcomes. As PD-1 aids in the proliferation and growth of tumor cells, a humanized monoclonal antibody pembrolizumab (MK-3475) was made to inhibit the PD-1 receptor. The NCT02313272 trial was conducted with WHO grade IV glioma patients by administrating pembrolizumab and bevacizumab (a humanized anti-VEGF monoclonal antibody) (Table 1). This trial examined the efficacy of pembrolizumab and bevacizumab with a combination of hypofractionated stereotactic radiotherapy (HFSRT) by enrolling 32 patients. It also developed a mathematical model from clinically observed longitudinal volumetric tumor growth. The tumor volume was accessed using T1-weighted MRI before treatment and all patients received consecutive HFSRT treatment based on the gross tumor volumes. In addition to radiotherapy, the VEGF inhibitor bevacizumab was injected into the patients intravenously every 2 weeks and pembrolizumab was infused into patients every 3 weeks. Out of 32 participants, only 16 patients were monitored for tumor progression, and analysis showed that bevacizumab played a role in reducing the incidence of cerebral edema and radiation-caused necrosis. The median progression-free survival among treated patients suggests that combination therapy can have better outcomes than single therapy. The NCT02529072 trial investigated the potential safety dose of nivolumab, which targets PD-1, and the efficacy to combine nivolumab with a DC vaccine. The trial was conducted based on two types of treatments, one with nivolumab monotherapy and the other with an intradermal infusion of a DC vaccine with nivolumab. The progression-free survival (PFS) for this trial was 6-48 months, whereas the median overall survival was 4 years. The treatment-related adverse effects due to systematic toxicities imply the safety profile of nivolumab administration is similar to that when combined with a DC vaccine. The NCT02798406 trial was conducted by investigating the efficiency and safety of a single intratumoral injection of pembrolizumab as an immune checkpoint inhibitor and DNX 2401, which is a genetically modified oncolytic adenovirus. With an enrollment of 49 patients diagnosed with glioblastoma, albeit one patient diagnosed with gliosarcoma, the primary endpoint of the trial measured the objective response rate, which was 10.4% and statistically different from a prespecified historical response rate of 5%. The median overall survival was 12.5 months and no dose-limiting toxicities were observed. With promising survival times, combinations of these two immune therapy agents appear to provide a better response toward recurrent glioblastoma and gliosarcoma patients. A phase 1 study from the NCT02852655 trial investigated the efficacy of neoadjuvant anti PD-1 (pembrolizumab) before surgical resection and treatment with adjuvant only. However, no results from this trial have been published. The NCT02667587 clinical trial was conducted to evaluate the efficacy of a PD-1 inhibitor nivolumab, in combination with radiation therapy and temozolomide, on newly diagnosed GBM patients with a *MGMT* promoter. It provides evidence that when patients who have methylated-MGMT promoters are treated with an alkylating agent such as temozolomide, the overall survival times are longer than those who have tumors with an unmethylated MGMT. The NCT02337686 trial was conducted to ascertain the efficacy of pembrolizumab with GBM patients. The median overall survival was 20.3 months and the PFS was 4.5 months. The NCT02550249 trial achieved a median overall survival of 7.3 months using nivolumab. The analysis of nivolumab-treated patients showed the upregulation of immune-related transcripts such as *CXCL10*, *CCL4*, and *CCL3L1,* and a downregulation of *CRP*, *SSX4*, and *CR2* targets. Another human IgG1 mAb against PD-L1 (durvalumab) was used in the NCT02336165 trial and the median overall survival was 15.1 months. The NCT02017717 trial examined the efficacy and safety of nivolumab vs. bevacizumab in patients with recurrent GBM. It was reported that patients with/without an *MGMT* promoter had a longer median overall survival in the nivolumab group than the bevacizumab group. However, this study did not meet the primary endpoint of overall survival. 

#### 3.1.3. T Cell Immunoglobulin and Mucin Domain 3 (TIM-3)

The co-inhibitory receptor TIM-3 is expressed on CD4^+^ and CD8^+^ T cells and plays a key role in the regulation of the immune response, including activation and the differentiation of T cells [54]. As a negative regulator of the lymphocyte function, the TIM-3 establishes T-cell exhaustion by suppressing their responses when interacting with its ligand [55]. It belongs to an immunoglobin (Ig) superfamily and is found in the T cells of both mice and humans [56]. The TIM-3 can recruit a ligand named galectin-9, which helps in the upregulation of intracellular calcium flux, leading to cell death via TIM-3 and galectin-9 mediated pathways [57,58,59,60,61,62]. As a TIM-3 ligand, galectin-9 (also known as S-type lectins) is expressed in lymphocytes and various cell types. Galectins are associated with the carbohydrate-binding protein family: a group of proteins that are connected to a critical role in regulating immune cell homeostasis and inflammation. This fact provides an intriguing paradigm in which IFN-γ can generate galectin-9 in the targeted cells, which can eradicate Th1 cells and hence prevent organ-specific chronic inflammation. The galectin-9 was upregulated in the CNS on day 10 after immunization to induce experimental autoimmune encephalomyelitis, at a time when T-cell infiltration and TIM-3 expression were at their highest. This is consistent with the observation that encephalitogenic T cells induce encephalomyelitis to produce IFN-γ, and undergo rapid cell death once activated in the brain. This could also be the start of a decrease in inflammation and remission from autoimmune illness [63,64]. By upregulating galectin-9, which in turn inhibits TH1-mediated inflammation, IFN-γ plays a role in both the pro-inflammatory and anti-inflammatory aspects of inflammation. Because IFN-γ deficiency would disrupt this regulatory loop, this may be the underlying mechanism causing the severe encephalomyelitis seen in IFN-γ-deficient mice. Ultimately, research demonstrates that the interaction between TIM-3 and galectin-9 serves as a mechanism to reduce immunity through the selective deletion of TIM-expressing T-helper (TH1) cells. The development of the TIM-3-galectin-9 pathway has been used to prevent chronic inflammation in target tissues and to regulate TH1 cell population expansion and tolerance in the immune compartment [65,66]. By a combination of TIM-3 antibody MBG453 (sabatolimab) and spartalizumab (a PD-1 checkpoint inhibitor) in the treatment of recurrent GBM, the phase 1 NCT03961971 trial is active, but no results have been posted yet (Table 1).

#### 3.1.4. Indoleamine 2, 3-Dioxygenase 1 (IDO-1)

**Figure 5:**
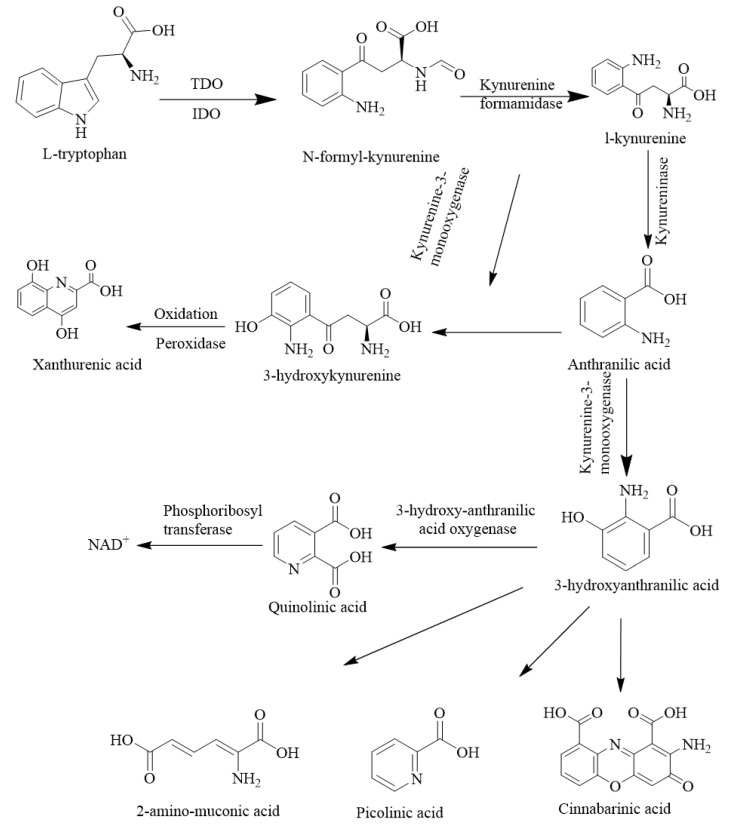
The different kynurenine-mediated pathways of indoleamine 2,3-dioxygenase 1 (IDO-1).

The indoleamine 2,3-dioxygenase 1 (IDO-1) is a checkpoint molecule found on GBM and immune cells, which can convert tryptophan to N-formyl kynurenine (NFK) and its eventual downstream catabolite kynurenine (KYN) by formamidase, and has since been implicated in immunosuppression [67]. The KYN is the main component that has the potential to trigger the KYN pathway. This pathway leads to the formation of three major catabolites, quinolinic acid, kynurenic acid, and picolinic acid, which are collectively named as KYNs for regulating the inhibition of T-cell proliferation, as well as causing apoptosis [68]. The IDO-1 expression was inhibited by 1-methyl tryptophan, which ultimately helps to protect the fetus from a T cell-mediated attack [69]. For gliomas, upregulation of IDO-1 is associated with immune suppression and the patient usually shows a reduced survival rate. The expression of IDO-1 is positively correlated with immunosuppressive regulatory T-cell infiltration and is negatively correlated with patient prognosis [70,71,72,73]. There is strong evidence supporting the overexpression of IDO-1 in human glial cell lines, as well as in human glioblastoma biopsies. The mechanisms of IDO-1 function, tryptophan depletion, or accumulation of tryptophan toxic metabolites, are shown in Figure 5. 

The IDO-1 activity in human glial cell lines is influenced by the production of IFN-γ and the activity of IDO-1 could be enhanced if IFN-γ is produced from activated T cells or neurons. Ozawa et al. studied the expression of IDO-1 at both the mRNA level and the protein level while emphasizing the role of IFN-β [74]. They found that other than IFN-γ, IFN-β also helps in increasing the expression of IDO-1 in glioma stem cells, causing treatment resistance. The PF-06840003 is an inhibitor of IDO-1, which has been applied in clinical trials for patients with recurrent malignant glioma, by which the inhibition of ^13^C_10_ kynurenine and endogenous kynurenine was successfully demonstrated [75,76,77]. Similarly, indoximod is an IDO-1 checkpoint inhibitor that can reverse the immunosuppressive effects of high KYN levels from the activity of IDO-1 [78]. It was used with pembrolizumab (a PD-1 antibody) for the treatment of advanced melanoma in a phase 2 trial [79]. The NCT02052648 trial involves the administration of indoximod as an IDO-1 inhibitor, with a chemotherapeutic drug, temozolomide, to treat patients suffering from temozolomide-refractory primary malignant brain tumors (Table 1). The NCTO4047706 trial examined the dose-limiting toxicity of BMS-986205 (an IDO-1 inhibitor) in combination with nivolumab and radiotherapy.

#### 3.1.5. Lymphocyte Activation Gene-3 (LAG-3)

The lymphocyte activation gene-3 (LAG-3) is a protein, encoded by the *LAG3* gene in humans, with 503 amino acid residues and a significant immune checkpoint. As a co-inhibitory receptor, its main characteristic function is to suppress T-cell activation and cytokine secretion by mediating a state of immune homeostasis [80]. In 1990, Triebel and his team first introduced LAG-3 and showed that it is closely related to CD4 in terms of chromosomal localization [81]. It is expressed on cell membranes of tumor-infiltrating lymphocytes (TILs), natural killer (NK) cells, dendritic cells (DCs), and B cells. It is expressed on CD4^+^/CD8^+^ double-positive cells, but it competes with CD4 to bind with major histocompatibility class II (MHC class II) molecules, thus downregulating the function of CD4 and promoting apoptosis. A study conducted by Maximilian and his group about the LAG-3 expression in human glioma suggested the diversity of immune microenvironment composition of glioma, and the combination of anti-PD-1 with LAG-3 checkpoint inhibitor is more effective at an earlier point [82]. Bookman et al. also showed LAG-3 to be an early marker of exhausted T cells [83]. The LAG-3 targeting therapies can be classified into three categories, anti-LAG-3 monoclonal antibody, bispecific LAG-3, and LAG-3 Ig fusion proteins. The anti-LAG-3 monoclonal antibody helps in cracking down the signals which reach the monocytes via MHC class II molecules, which simultaneously delays the T-cell response to IL-12 and leads to the blockade of LAG-3/MHC contacts [84]. The bispecific LAG-3 confines with two binding sites, which can target two different antigens or epitopes on the same antigen [85]. The clinical applications of LAG-3 in GBM patients are very limited. A phase 1 clinical trial NCT02658981 showed that using anti-LAG-3 monoclonal antibody BMS-986016 or BMS-663513 (anti-CD137) can improve the survival rate in patients with recurrent GBM, when combined with anti-PD-1 [86] (Table 1). Undoubtedly, the molecular mechanism and signaling pathways of LAG-3 interacting with other checkpoints still need to be elucidated. 

### 3.2. Vaccines

Vaccines are a class of immunotherapies that can help to induce the activity of certain immune-based antigens, non-tumor-specific antigens (NTSAs), tumor-associated antigens (TAAs) or tumor-specific antigens (TSAs). Generally, TSAs that comprise mutant proteins expressed from tumors are preferred over TAAs due to their selective expression on the localized tumor site [87]. For brain tumors, tumor-specific T-cell activation may counteract malignant brain tumor recurrence and progression [88]. By inducing an anti-tumor immune response, the dendritic cell vaccination represents an active immunotherapy in the treatment of GBM [89]. Several examples of experimental cancer vaccines have been reported, including allogeneic and autologous tumor cells, tumor lysates, synthetic peptides, proteins, antigen-loaded dendritic cells, “naked” DNA, and recombinant viral vectors [90,91,92]. The clinical trials taken from clinicaltrials.gov using vaccines (dendritic cell-based vaccine, peptide-based vaccine, or viral-based vaccine) for GBM treatment are included in Table 2.

#### 3.2.1. Dendritic Cell-Based Vaccines

Dendritic cells (DCs) are the immune system’s most powerful antigen-presenting cells. They serve as the immune system’s sentinels in their immature form, constantly searching the surroundings for antigens. After capturing the protein antigens by DCs through internalized endocytosis, fragmentation of the proteins will lead to the generation of peptides in the endosomal/lysosomal compartments [93]. Exogenous antigens are presented as antigenic peptides on the cell surface in MHC class II complexes, whereas endogenous antigens are expressed in MHC class I. The DCs have the unusual capacity to present internalized antigens that originated from external sources, not only in MHC class II molecules, but also in MHC class I molecules, in a process known as cross-presentation [94]. Tumor antigens, for example, can be given to CD8^+^ T lymphocytes in this way. In a mature stage, DCs migrate to lymphoid organs and offer the antigen to naive T lymphocytes. Activated T cells then multiply and exit the lymph nodes to go on quest and kill cancer cells in an antigen-dependent manner. As a diverse cell population, the natural DCs in human peripheral blood can be classified into two types, myeloid DCs (mDCs) and plasmacytoid DCs (pDCs). Human mDCs are further classified into two groups, blood dendritic cells BDCA-1 and BDCA-3, based on the differential surface expression of CDc1 (conventional Dc1) [95]. Natural DC subtypes vary from each other in several ways, such as function, organelle localization, and phenotype. Both mDCs and pDCs express specific Toll-like receptors (TLRs) and display different responses toward pathogenic stimuli, indicating that each group has a specific purpose in guiding immune responses. Following detection and controlling viral infection, pDCs produce high levels of type 1 IFN. The DC-based vaccine was first clinically implemented in 1996 on patients with B cells lymphoma [96]. For brain tumors, the NCT02529072 clinical trial investigated the DC vaccine cytomegalovirus pp65 lysosomal associated membrane protein (CMVpp65) against GBM (Table 2). In this trial, an anti-PD-1 monoclonal antibody (nivolumab) was combined with CMVpp65 in the phase 1 trial to enhance the efficacy of the DC vaccine. Recently, the NCT00045968 reported a phase 3 trial of an autologous tumor lysate-loaded dendritic cell vaccine (DCVax-L) against patients with recurrent and newly diagnosed GBM. With an enrollment of 331 patients, the patients were randomized to DCVax-L or placebo plus standard-of-care adjuvant temozolomide groups [97]. About 2.5 million dendritic cells were loaded in each DCVax-L dose and were administrated intradermally in the upper arm of the patients. The median overall survival of newly diagnosed GBM patients receiving DCVax-L was 19.3 months vs. 16.5 months for patients receiving this standard of care. The median survival time also extended from 7.8 months to 13.2 months for recurrent GBM patients. Overall, the DCVax-L resulted in the clinically meaningful extension of survival for patients with both types of GBM.

#### 3.2.2. Peptide-Based Vaccine 

Peptide-based vaccines are generally composed of TAAs and TSAs for better efficacy of the vaccine. To elicit the response of CD4^+^ and CD8^+^ T cells, mixing peptides with adjuvants can have a better impact on tumor cells, as the absence of adjuvants may influence the delivery of antigens. The peptide-based vaccines are usually comprised of nine amino acids that can bind to the distinctive MHC class I antigen [98]. To make a vaccine, a peptide tailored to individual patients can be combined with an adjuvant and administered subcutaneously every 7 to 14 days. After collecting the injected peptide by antigen-presenting cells (APCs), the complex migrates to regional lymph nodes and offers the loaded peptides to circulating cytotoxic T lymphocytes (CTLs). The CTLs can recognize a peptide on APCs and be activated in conjunction with clonal proliferation in the nodes. These activated CTLs exit the lymph nodes or blood circulation, travel and infiltrate into tumor locations, detect the appropriate peptide-MHC complex on cancer cells, and subsequently destroy cancer cells and therefore lead to tumor regression [99]. The epidermal growth factor receptor variant III (EGFRvIII) vaccine was formulated by a modified keyhole limpet hemocyanin (KLH) peptide that can drive tumorigenesis. The mutation burden of GBM is generally modest; yet, tumor heterogeneity remains a challenge, particularly for selective single-target therapy [100,101]. Antigen escape, where the tumor no longer expresses the target antigen, can also impede the treatment for GBM [102]. As a result, it is critical to construct a model that can recognize new antigens and forecast HLA presentation capabilities. Two recent trials have emphasized the development of tailored cancer vaccinations against new antigens. In the first investigation, a tailored cancer vaccination against a new antigen was produced by comparing entire exon sequence data from resected tumors and matching them with normal tissues. For each patient, 7 to 20 antigens were chosen for vaccine development, which were projected to have a high affinity for HLA type-1 binding [103]. To enhance the number of binding epitopes, the second study coupled two novel antigens with non-mutated tumor-associated antigens. After injection, nine non-mutated peptides were added to a vaccine formulation in the glioma actively personalized vaccine consortium 1 (APVAC1) vaccine, followed by the addition of 20 additional antigen peptides in the APVAC 2 vaccine. Both the phase 1 clinical trials were reported to be capable of inducing a significant number of invasive tumor-reactive T cells, as well as a clonal proliferation of antigen-specific cells. The rindopepimut is another peptide-based vaccine with a 14-mer peptide, which targets epidermal growth factor receptor variant III (EGFRvIII), a mutant variant of EGFR found in GBM patients [104]. 

#### 3.2.3. Viral-Based Vaccine 

Viral-based therapeutics for glioma treatment represent an alternative immunization strategy since they help with the trafficking of pathogen-associated molecular patterns, which are responsible for pathogenic infections and enhancing tumor-antigen secretion from dying virus-infected cells. The viral-based therapy can be thought of as an in situ immunization of the tumor, including the entire repertory of neoantigens. Furthermore, this technique has the potential to convert an immunosuppressive tumor into a “hot” tumor that is capable of eliciting Th1-primed T-cell responses. Promising engineered oncolytic viruses in clinical trials for the treatment of patients with high-grade gliomas, including herpes virus-1 (HSV-1G47Δ), adenovirus DNX-2401 (or Delta24-RGD), and poliovirus-rhinovirus chimera PVS-RIPO [105,106,107]. 

The oncolytic adenovirus DNX-2401 replicates only in tumor cells. A 24-base pair deletion in the E1A gene, coding for a protein that interacts with the retinoblastoma tumor suppressor protein, distinguishes this genetically altered virus [108]. Although the recombinant virus can reproduce within tumor cells, it cannot multiply within normal somatic cells. Furthermore, the virus receptor can be modified with an Arg-Gly-Asp (RGD) tripeptide that targets glioma-related integrin, allowing for targeted viral entrance into tumor cells. The phase 1/2 clinical trial NCT01582516, with 20 patients, intends to determine the safety of Delta-24-RGD in patients with recurrent GBM by administrating the virus to the tumor and the surrounding infiltrated brain with convection-enhanced delivery, but no results have been posted. However, a study published last year reveals the convection-enhanced delivery of the oncolytic adenovirus Delta24-RGD to 20 patients with recurrent GBM provides a safe treatment in this phase 1 trial, with increased numbers of macrophages and CD4^+^ and CD8^+^ T cells found in the tumor specimens [109]. In the phase 1 clinical trial of DNX-2401 (Delta24-RGD), 20% of the 37 patients with recurrent malignant glioma showed more than three years of progression-free survival from the time of treatment after receiving the viral vaccine. In tumor specimens collected from the patients, the DNX-2401 was found to replicate and spread within the tumor, indicating direct virus-induced oncolysis, but inflammation was found from radiography. Immune cell infiltration by CD8^+^ and CD4^+^ T cells, that express the Th1-specific transcription marker, T-bet, was found, together with the downregulation of the transmembrane immunoglobulin mucin-3 (TIM-3) in the histopathologic examination of the specimen [110]. The mesenchymal stem cells (MSCs) can be a novel delivery vehicle for the treatment of metastatic malignancies and isolated tumors with their tumor-trophic migration characteristics [111]. Therefore, employing viruses released from MSCs is a promising anti-cancer treatment modality addressing to a variety of cancers. The glioblastoma treatment with DNX-2401 in a murine model was successful by using a fibrin scaffold for transplanting DNX-2401-loaded MSCs after surgical resection [112]. For humans, the DNX-2401 is currently being studied in a new phase 1 clinical trial (NCT03896568), which is delivered to recurrent GBM patients via intra-arterial injection of DNX-2401-loaded allogeneic human bone marrow MSCs, rather than via intratumoral injection. A phase 2 trial using replicative adenovirus DNX-2401, with a PD-1 antibody pembrolizumab for recurrent glioblastoma or gliosarcoma, was reported in NCT02798406.

The excellent oncolytic capacity of poliovirus PVS-RIPO coincides with its unique ability to bind with a poliovirus receptor (PVR, CD155) expressed in human glioma cells. To minimize neurotoxicity, the internal ribosome entry site was substituted with the nonvirulent human rhinovirus type 2 (HRV2) [113]. The NCT01491893 trial was conducted by intratumoral injection of PVS-RIPO in a phase 1 clinical trial involving 61 patients with recurrent GBM (Table 2). From magnetic resonance imaging, inflammation in the GBM patients was found together with pseudoprogression. However, no other serious side effects were noted, and the patient’s overall survival rate was 21% at 36 months, which was higher than that in the control group at 4% after 36 months. This phase 2 study is currently underway for further investigation of the PVS-RIPO viral treatment.

#### 3.2.4. Delivery of Cancer Vaccine by Nanomaterials 

Considering the delivery of cancer vaccines, the delivery using nanomaterials has garnered excitement from researchers due to its immune-stimulating nature against strong antigen-specific immune responses. To elicit effective and long-lasting benefits, the vaccine immune response can be tuned by optimizing the physicochemical properties of the nanocarrier or by modifying them with targeting molecules, as well as by co-encapsulating immunostimulators with the nanomaterials [114]. Various kinds of nanomaterial-based approaches have reported positive outcomes, due to the small size of the vehicle, low toxicity, and better permeability, which can help in overcoming the systematic biological barriers to induce better cellular immune responses [115]. Cationic nanoparticles are the best delivery vehicles within the nanomaterials to achieve better immunogenicity. The composition of this type of vaccine can be classified based on the types of antigens, the immune-stimulating adjuvants, additional excipients, and the cationic nanoparticle components. A direct comparison of three cationic nanoparticles (liposomes, chitosan-coated PLGA, and maltodextrin-based nanoparticles) to their anionic equivalents showed superior intracellular protein delivery for these cationic nanoparticles to induce strong cellular immune responses when exposed to DCs [116]. The in vivo models also indicate that cationic nanoparticles are efficient delivery vehicles for mRNA-based vaccines [117]. The incubation of murine bone marrow-derived DCs with DOTAP-containing liposomes can lead to the induction of several chemokines mediated by the extracellular signal-regulated kinase (ERK) pathway [116]. However, even though lipid-based nanoparticles serve as better drug carriers to the TME, no successful clinical applications have been reported yet. 

### 3.3. Chimeric Antigen Receptors T (CAR T) Cells 

**Figure 6:**
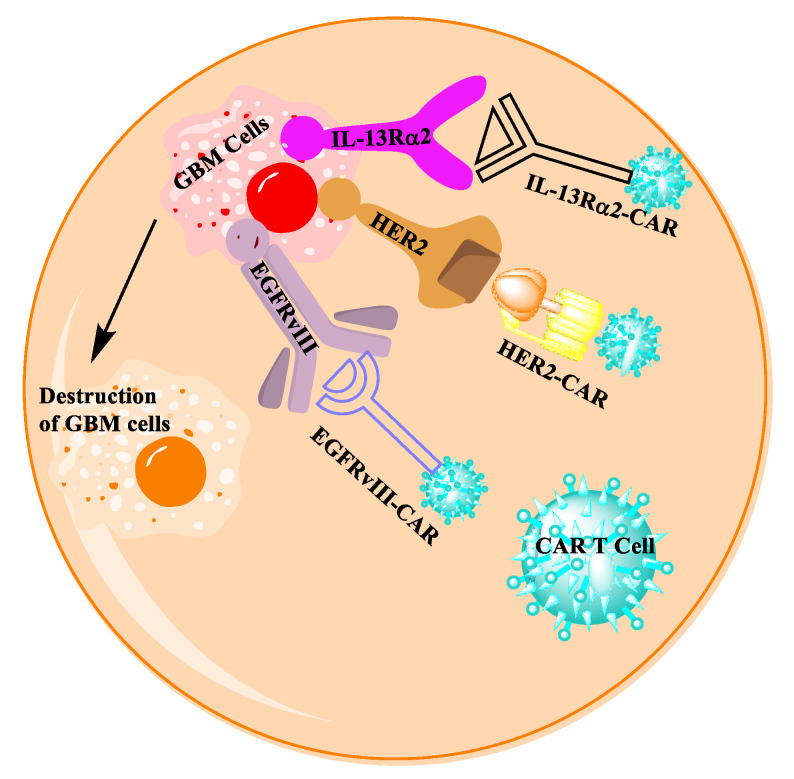
The mechanism that chimeric antigen receptors T (CAR T) cells play in GBM treatment.

The concept of chimeric antigen receptors T (CAR T)-cell therapy confines to T-cell activation by engineering T cells to express a synthetic receptor that can bind specifically to an antigen overexpressed on the cancer cell surface [118]. By inserting the gene for a special CAR receptor into the T cells, the CAR T cells (EGFRvIII-CAR, IL13Rα2-CAR, or HER2-CAR) can kill cancer cells by binding to the antigen, EGFRvIII, IL13Rα2, or HER2, that is overexpressed on GBM cells (Figure 6). By inducing the release of cytokines during T-cell activation, cytokines can be localized at the target antigen to lessen the inflammation. On the other hand, patients treated with CAR T therapy may also develop inflammation leading to cytokine release syndrome [119]. Neurogenic disorders like aphasia and delirium may also occur from adverse effects arising from the presence of a large amount of cytokines. In GBM patients, the expression of epidermal growth factor receptor variant III (EGFRvIII) is higher, which is a mutation form of EGFR. The clinical trial NCT01454596 was conducted with GBM patients exhibiting a high EGFRvIII expression. The patients were infused with EGFRvIII-CAR cells based on the percentage of expression of EGFRvIII (71% median value through MRI database). The outcome of the phase 1 study suggests no adverse effect, such as neurotoxicity, and demonstrates the target specificity of CAR T cells. After EGFRvIII, the major cancer antigen expressed in almost 60% of GBM patients is interleukin-13 receptor alpha chain variant 2 (IL13Rα2) [120]. This antigen plays a major role in the activation of phosphatidylinosinositol-3 kinase (PI-3K)/AKT/mammalian target of the rapamycin (mTOR) pathway [121]. The IL13Rα2 antigen can be targeted with engineered autologous CD8^+^ T cells. After injecting CAR T cells into the tumor cavities, a reduction in tumor volume was noted, implying the antitumor activity of IL13Rα2-CART cells. However, due to the heterogeneity of IL13Rα2 expression, one patient infused with both an intraventricular and intracavity CAR T-cells injection showed regression of all intracranial and spinal tumors [122]. The active NCT02208362 trial reported results from 82 patients in this phase 1 study. The NCT01082926 phase 1 trial-injected GRm13Z40-2, CAR CD8^+^ T cells, expressed an IL13 zetakine/herpes-simplex virus 1 thymidine kinase fusion (HyTk). The patients with unresectable recurrent/refractory GBM were treated in conjunction with IL2 and repetitive doses of CAR T cells. This trial demonstrated minimal side effects from the anti-glioma response in patients with the IL13Rα2-expressing GBM. The human epidermal growth factor receptor-2 (HER2) belongs to the family of tyrosine kinase receptors, which binds to a subset of phosphotyrosine-binding proteins. The HER2 does not bind to any ligands, but preferably binds with the ligands of other members of the receptor family, such as HER1, HER3, and HER4 [123]. The NCT01109095 trial was conducted to investigate the anti-glioblastoma activity of HER2-CART in virus-specific T cells in 17 patients. The median survival of this clinical study was 11.1 months after CAR T-cell infusion (Table 1). The clinical trials taken from clinicaltrials.gov using CAR T cells, as an immune-based therapeutic approach for GBM, are included in Table 3.

### 3.4. Natural Killer (NK) Cells 

**Figure 7:**
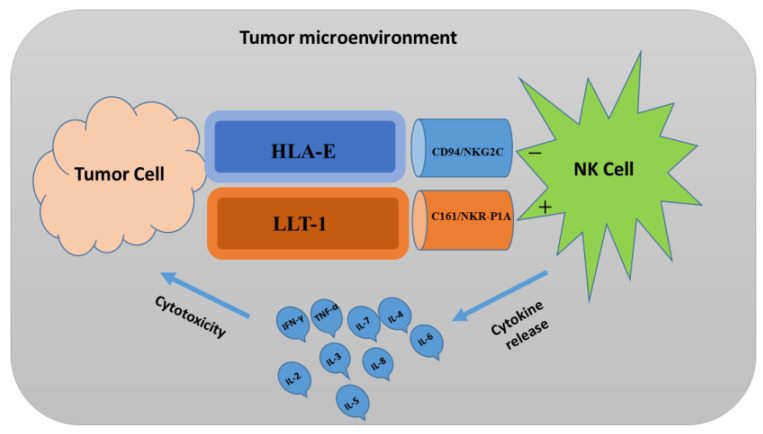
The regulation of natural killer (NK)-cell activation through activating (+) and inhibitory (−) receptor signaling. The tumor cells may lose their MHC class I molecule HLA-E, a ligand for inhibitory receptor NKG-2C on NK cells, and at the same time acquire a stress-associated molecule such as LLT-1, which acts as a ligand for the activating receptor NKR-P1A on NK cells. The lack of inhibitory signaling, coupled with the induction of activation signaling, shifts the balance toward NK-cell activation, leading to the secretion of pro-inflammatory cytokines, such as IFN-γ, TNF-α, and interleukins (ILs), and cytotoxicity toward tumor cells.

As large granular lymphocytes, the natural killer (NK) cells play an important role in tumor immune surveillance. With the help of various sets of germline-encoded surface receptors, such as pathogen-recognition receptors, NK cells can detect and functionalize on malignant cells without prior sensitization of the host. Similar to the activated cytotoxic T cells, the activation of NK cells can release cytotoxic granules comprising lysosome and late-endosome components that have different proportions of the dense-core domain, in which perforin and granzymes are stored [124]. During an immune response, NK cells produce chemokines, as well as cytokines, including interferon-gamma (IFN-γ) and tumor necrosis factor-alpha (TNF-α), which are important for host protection against viral infection and tumor formation [125]. The IFN-γ has the potential to induce target cell resistance to NK cytolysis [126]. The stimulation of NK cells depends upon the integration of signals derived from both the activating and inhibitory receptors on their surface. Compared with normal cells, the tumor cells have down-regulated MHC class I molecule expression, which can be recognized by NK cell inhibitory receptors, leading to lower inhibitory signal in NK cells [127]. Therefore, the signal from activating receptors in NK cells leads to NK-cell activation for the elimination of tumor cells either directly by NK cell-mediated cytotoxicity or indirectly through the secretion of pro-inflammatory cytokines [127]. In humans, the NK cells can overexpress a homodimer-inhibitory receptor: NKR-P1A (CD161). By binding with the NKR-P1A, the ligand lectin-like transcript-1 (LLT1) can inhibit the NK cell-mediated cytotoxicity (Figure 7) [128]. Alternatively, as a major component present in the immune system in human leukocyte antigen (HLA), the HLA class I histocompatibility antigen, alpha chain E (HLA-E) can act as an immune checkpoint in the activation of the NK cells via interaction with the activating receptor NKG-2C (CD94) of NK cells. As far as NK-cell therapies are concerned, the most challenging aspect of GBM treatment has been the immunosuppressive TME of brain tumors, which interferes with the activation of NK cells by overriding inhibition at the same time [129]. The clinical trial taken from clinicaltrials.gov, using NK cells for GBM treatment, is included in Table 4.

### 3.5. Biomarkers 

In the interpretation of cell-based strategies for all cancers, one of the biggest obstacles to date is detection and tracking. The tumor biopsy-based techniques for evaluating the responses, such as immunohistochemistry and flow cytometry remain worthless and are often limited in their use for tumor prognosis, as well as treatments, leading to poor outcomes in clinical trials. For GBM, the significant genes which drive the TME are phosphatase and tensin homologue (*PTEN*), tumor protein 53 (*TP53*), platelet-derived growth factor receptor-α (*PDGFRA*), neurofibromatosis type 1 (*NF1*), telomerase reverse transcriptase promoter (*TERTp*), and epidermal growth factor receptor (*EGFR*) [130,131]. Undoubtedly, the prognosis and treatment of GBM patients require different kinds of biomarkers. For example, clinically, the hemodynamic multiparametric tissue signature (HTS) biomarker can describe the heterogeneity of tumors based on the angiogenic process at tumor regions of GBM patients. The clinical trial NCT03439332, with 305 participants, intends to determine if preoperative vascular heterogeneity of glioblastoma is predictive of the overall survival of patients undergoing standard-of-care treatment by using the hemodynamic multiparametric tissue-signature (HTS) biomarker (Table 5). The study found that a high impact of MGMT status in patients with moderately vascularized tumors and the characterization of tumor vascularization may help to improve the estimation of responsiveness to standard-of-care treatment (temozolomide) and prognosis, rather than MGMT methylation assessment alone. There is a growing interest to use O^6^-methylguanine DNA methyltransferase (MGMT) and isocitrate dehydrogenase 1 (IDH1) as prognostic or predictive biomarkers in the clinical setting, and their indications are based on the standard-of-care guidelines [132]. The *MGMT* gene is responsible for a DNA repair enzyme that can help to extricate tumor cells from damage that is induced by alkylating agents. The DNA methylation results in the silencing of this gene through interaction with the sequence-specific binding of positive transcription factors. Studies using MGMT methylation as a prognostic and predictive biomarker of GBM were reported before, in which a statistically significant difference in the level of DNA methylation from 5-methylcytosine content was found when compared with normal tissue and benign neoplasms [133,134]. The DNA methyltransferase, which is encoded by the promoter of MGMT, prevents the mutation of the glycine C-acetyltransferase (*GCAT*) gene caused by alkylating agents such as temozolomide (TMZ) and lomustine (CCNU) [135]. The MGMT promoter, methylation, is associated with outcomes in GBM patients treated with TMZ and radiation therapy, versus radiation therapy alone, in TMZ-treated patients [136]. For MGMT methylation studies conducted in GBM patients after immunotherapy, this biomarker manifests itself as a strong prognostic GBM biomarker in clinical practice [137,138]. Circulatory biomarkers associated with anti-VEGF therapy are also useful to obtain effective treatments and responses in GBM patients [139]. According to the database evaluated by The Cancer Genome Atlas, PD-1 and PD-L1 are categorized as poor prognostic biomarkers [140]. For imaging biomarkers, so far, there are no clinically approved ones. However, to distinguish the phenotypes of GBM, different imaging techniques other than MRI, such as positron emission tomography (PET), diffusion-weighted magnetic resonance imaging (DW-MRI) with apparent diffusion-coefficient mapping (ADC) and dynamic susceptibility-weighted contrast-enhanced perfusion imaging, are predictive biomarkers for GBM patients [141].

## 4. Conclusions and Outlook

In recent years, intense research efforts emphasize rerouting and activating adaptive immune responses to treat glioblastoma. Nearly all clinical studies using immune checkpoint blockade and vaccination show encouraging signs of immune responses, which partially increase overall survival. However, barriers to effective treatment still exist. These include intrinsic limiting elements associated with tumor biologies, such as the immunosuppressive microenvironment of GBM, the potential deficiency in T-cell homing, and the tumor heterogeneity in GBM, coupled with insufficient clonal neoantigens. The frequent use of glucocorticoids, the insufficient intratumoral bio-distribution of therapeutic antibodies (such as anti-PD-1 and anti-PD-L1), and the induction of peripheral immunity are additional factors that restrict the efficacy of immunotherapy in treating GBM. Studying the different phenotypes of GBM and standard of care, combined with improved immunotherapies, may increase the overall survival of patients. Continued efforts should concentrate on underpinning individual treatments to show the biological pathways involved in immunosuppression and to overcome the barriers. Although immune checkpoint inhibitor drugs have shown the most promising results within all immunotherapeutic approaches in GBM treatment, many patients do not respond to immunotherapies, and some, over time, can develop a resistance to such treatment. With complex mechanisms behind tumor immune resistance, many of them are well characterized, but many are still unknown. With this limitation and challenge in GBM immunotherapy, there is an urgent need in GBM or other cancer immunotherapy to elucidate the complex drug-resistant mechanisms involved, and thus develop an effective combination therapeutic approach for overcoming the hurdle in GBM treatment.

## Figures and Tables

**Table 1:** The clinical trial examples using immune checkpoint inhibitors for GBM treatment from clinicaltrials.gov, accessed on 9 January 2023.

Method	Identifier	Title	Patients	Phase	Treatment	Status
CTLA-4	NCT02794883	A phase 2, open label, clinical trial of pre surgical and adjuvant treatment of recurrent malignant glioma with tremelimumab and durvalumab (MEDI4736) alone and in combination to determine immunologic changes from treatment	36	2	Tremelimumab Durvalumab	Completed
PD-1	NCT02667587	A randomized phase 3 single-blind study of temozolomide, plus radiation therapy combined with nivolumab or placebo in newly diagnosed adult subjects with MGMT-methylated glioblastoma	716	3	Nivolumab Temozolomide	Active
	NCT02313272	Hypofractionated stereotactic irradiation with pembrolizumab and bevacizumab in patients with recurrent high grade gliomas	32	1	Pembrolizumab Bevacizumab	Completed
	NCT02529072	Nivolumab with DC vaccines for recurrent brain tumors	6	1	Nivolumab DC vaccine	Completed
	NCT02798406	Combination adenovirus + pembrolizumab to trigger immune virus effects	49	2	Pembrolizumab Adenovirus	Completed
	NCT02852655	A pilot surgical trial to evaluate early immunologic pharmacodynamic parameters for the PD-1 checkpoint inhibitor, pembrolizumab (MK-3475), in patients with surgically accessible recurrent/progressive glioblastoma	25	1	Pembrolizumab	Completed
	NCT02337686	Pharmacodynamic study of pembrolizumab in patients with recurrent glioblastoma	18	2	Pembrolizumab	Active
	NCT02550249	Phase 2 study of neoadjuvant nivolumab in patients with glioblastoma multiforme	29	2	Nivolumab	Completed
	NCT02336165	Phase 2 study to evaluate the clinical efficacy and safety of durvalumab (MEDI4736) in patients with glioblastoma (GBM)	159	2	Durvalumab	Completed
	NCT02017717	A randomized phase 3 open label study of nivolumab versus bevacizumab and multiple phase 1 safety cohorts of nivolumab or nivolumab in combination with ipilimumab across different lines of glioblastoma	529	3	Nivolumab Bevacizumab Ipilimumab	Completed
	NCT02617589	A randomized phase 3 open label study of nivolumab vs. temozolomide each in combination with radiation therapy in newly diagnosed adult subjects with unmethylated MGMT (tumor o-6-methylguanine DNA methyltransferase) glioblastoma	560	3	Nivolumab Temozolomide	Completed
	NCT03452579	A randomized phase 2 open label study of nivolumab plus standard dose bevacizumab versus nivolumab plus low dose bevacizumab in recurrent glioblastoma (GBM)	90	2	Nivolumab Bevacizumab	Active
	NCT02337491	Phase 2 study of pembrolizumab (MK-3475) with and without bevacizumab for recurrent glioblastoma	89	2	Pembrolizumab Bevacizumab	Completed
	NCT02054806	Phase 1B study of pembrolizumab (MK-3475) in subjects with select advanced solid tumors	477	1	Pembrolizumab	Completed
TIM-3	NCT03961971	A phase I trial of anti-TIM-3 in combination with anti-PD-1 and stereotactic radiosurgery in recurrent GBM	16	1	MBG453 Spartalizumab	Active
IDO-1	NCT02052648	A phase 1/2 study of the combination of indoximod and temozolomide for adult patients with temozolomide-refractory primary malignant brain tumors	48	1/2	Indoximod Temozolomide	Completed
	NCT04047706	Combination of checkpoint inhibition and IDO1 inhibition together with standard radiotherapy or chemoradiotherapy in newly diagnosed glioblastoma. A phase 1 clinical and translational trial	30	1	BMS-986205	Active
LAG-3	NCT02658981	A phase 1 trial of anti-LAG-3 or anti-CD137 alone and in combination with anti-PD-1 in patients with recurrent GBM	63	1	BMS 986016	Active

**Table 2:** The clinical trial examples using vaccines for GBM treatment from clinicaltrials.gov, accessed on 9 January 2023.

Method	Identifier	Title	Patients	Phase	Treatment	Status
Dendritic cell (DC)-based vaccine	NCT02529072	AVeRT: Anti-PD-1 monoclonal antibody (nivolumab) in combination with DC vaccines for the treatment of recurrent grade 3 and grade 4 brain tumors	6	1	Nivolumab DC vaccine	Completed
	NCT00045968	A phase 3 clinical trial evaluating DCVax-L, autologous dendritic cells pulsed with tumor lysate antigen for the treatment of glioblastoma multiforme	348	3	DCVax-L	Active
	NCT03548571	Open label randomized phase 2/3 trial of dendritic cell immunotherapy against cancer stem cells in glioblastoma patients receiving standard therapy (DEN-STEM)	60	2/3	DEN-STEM	Active
	NCT00639639	Anti-tumor immunotherapy targeted against cytomegalovirus in patients with newly diagnosed glioblastoma multiforme during recovery from therapeutic temozolomide-induced lymphopenia	42	1/2	CMV-DC	Completed
Peptide-based vaccine	NCT00458601	A phase 2 study of CDX-110 with radiation and temozolomide in patients with newly diagnosed GBM	82	2	Rindopepimut CDX 110	Completed
	NCT01480479	An international randomized double, blind, controlled study of rindopepimut/GM-CSF with adjuvant temozolomide in patients with newly diagnosed, surgically resected, EGFRvIII-positive glioblastoma	745	3	Rindopepimut CDX 110	Completed
	NCT02454634	Targeting IDH1R132H in WHO grade 3–4 IDH132H mutated gliomas by a peptide vaccine-a phase 1 safety tolerability and immunogenicity multicenter trial (NOA 16)	39	1	IDH1 peptide NOA 16	Completed
Viral-based vaccine	NCT01582516	A phase 1/2 trial of a conditionally replication-competent adenovirus (Delta-24-RGD) administered by convection enhanced delivery in patients with recurrent glioblastoma	20	1/2	Delta-24-RGD	Completed
	NCT00805376	Phase 1 trial of conditionally replication-competent adenovirus (DNX-2401 formerly known as Delta-24-RGD-4C) for recurrent malignant gliomas	37	1	DNX-2401	Completed
	NCT03896568	Phase 1 clinical trial of allogeneic bone marrow human mesenchymal stem cells loaded with a tumor selective oncolytic adenovirus, DNX-2401, administered via intra-arterial injection in patients with recurrent high-grade glioma	36	1	DNX-2401	Recruiting
	NCT02798406	A phase 2 multi-center, open-label study of a conditionally replicative adenovirus DNX-2401 with pembrolizumab (KEYTRUDA^®^) for recurrent glioblastoma or gliosarcoma	49	2	DNX-2401	Completed
	NCT01491893	Dose-finding and safety study of an oncolytic polio/rhinovirus recombinant against recurrent WHO grade 4 malignant glioma	61	1/2	PVS-RIPO	Completed

**Table 3:** The clinical trial examples using chimeric antigen receptors T (CAR T) cells for GBM treatment from clinicaltrials.gov, accessed on 9 January 2023.

Method	Identifier	Title	Patients	Phase	Treatment	Status
CAR T cells	NCT01454596	A phase 1/2 study of the safety and feasibility of administering T cells expressing Anti-EGFRvIII chimeric antigen receptor to patients with malignant gliomas expressing EGFRvIII	18	1/2	EGFRvIII-CARs	Completed
	NCT02208362	Genetically modified T-cells in treating patients with recurrent or refractory malignant glioma	82	1	IL13Rα2-CARs	Active
	NCT01082926	Phase 1 study of cellular immunotherapy for recurrent/refractory malignant glioma using intratumoral infusions of GRm13Z40-2, an allogenic CD8^+^ cytolitic T-cell line genetically modified to express the IL13-zetakine and HyTK and to be resistant to glucocorticoids in combination with interleukin-2	6	1	GRm13Z40-2	Completed
	NCT01109095	Administration of HER2 chimeric antigen receptor expressing CMV-specific cytotoxic T cells in patients with glioblastoma multiforme (HERT-GBM)	16	1	HER2-CARs	Completed

**Table 4:** The clinical trial examples using natural killer (NK) cells for GBM treatment from clinicaltrials.gov, accessed on 9 January 2023.

Method	Identifier	Title	Patients	Phase	Treatment	Status
NK cells	NCT05108012	The safety evaluation of ex vivo activated haploidentical natural killer cells (NK) in recurrent glioblastoma multiform patients (clinical trial phase 1)	5	1	NK cells	Recruiting

**Table 5:** The clinical trial examples using biomarkers for GBM treatment from clinicaltrials.gov, accessed on 9 January 2023.

Method	Identifier	Title	Patients	Phase	Treatment	Status
Biomarkers	NCT03439332	Multicentre validation of hemodynamic multiparametric tissue signature (MTS) biomarkers from preoperative and postradiotherapy MRI in patients with glioblastoma: predictors of overall survival	305	Not available	HTS biomarker	Completed

## Data Availability

The data presented in this study are available on request from the corresponding author.
